# Multiple Mobile mRNA Signals Regulate Tuber Development in Potato

**DOI:** 10.3390/plants6010008

**Published:** 2017-02-10

**Authors:** David J. Hannapel, Anjan K. Banerjee

**Affiliations:** 1 Plant Biology Major, 253 Horticulture Hall, Iowa State University, Ames, IA 50011-1100, USA; 2 Biology Division, Indian Institute of Science Education and Research, Dr. Homi Bhabha Road, Pashan, Pune 411008, India; akb@iiserpune.ac.in

**Keywords:** phloem, signals, *Solanum tuberosum*, StBEL, TALE, tuberization

## Abstract

Included among the many signals that traffic through the sieve element system are full-length mRNAs that function to respond to the environment and to regulate development. In potato, several mRNAs that encode transcription factors from the three-amino-loop-extension (TALE) superfamily move from leaves to roots and stolons via the phloem to control growth and signal the onset of tuber formation. This RNA transport is enhanced by short-day conditions and is facilitated by RNA-binding proteins from the polypyrimidine tract-binding family of proteins. Regulation of growth is mediated by three mobile mRNAs that arise from vasculature in the leaf. One mRNA, *StBEL5*, functions to activate growth, whereas two other, sequence-related StBEL’s, *StBEL11* and *StBEL29*, function antagonistically to repress StBEL5 target genes involved in promoting tuber development. This dynamic system utilizes closely-linked phloem-mobile mRNAs to control growth in developing potato tubers. In creating a complex signaling pathway, potato has evolved a long-distance transport system that regulates underground organ development through closely-associated, full-length mRNAs that function as either activators or repressors.

## 1. Introduction

### 1.1. Phloem-Mobile mRNAs in Plants 

Plants have evolved a unique long-distance signaling system that utilizes cell-to-cell plasmodesmatal connections and a specialized phloem cell network. In addition to its function in the transport of sugars from source to sink organs, the phloem is an important conduit for moving signals that mediate responses to stress and processes of development [1]. Catalogued among the many signals that are transported across the sieve element system are metabolites, hormones, proteins, small RNAs and full-length mRNAs. Phloem sap profiling has confirmed that the phloem functions in a dynamic process to deliver signals that respond to internal and environmental cues. Numerous full-length mRNAs have been identified in the sieve element system of several plant species [2,3,4,5,6]. Using heterografts and movement assays, several mRNAs have been identified that are transported through the vascular system. Included in this ever-growing list are *Aux/IAA*, *FT*, *ATC*, *GAI* and *KNOX* (Table 1). Using a heterograft system between different plant species and RNA-Seq, Notaguchi et al. [7] identified 138 transcripts of *Arabidopsis* that were mobile across an *Arabidopsis*/tobacco graft union. Approximately ten of these were transcription factors (TFs), including transcripts from a BEL1-like and a KNOX-type TF. In another study, using variant ecotype transcript profiling in heterografts, Thieme et al. [8] reported the identification of 2006 genes producing mobile RNAs in *Arabidopsis*. Many of these mobile transcripts followed the phloem-dependent sugar allocation pathway from leaves to roots, but a high number of transcripts also moved in a root-to-shoot direction. Yet, despite these valuable insights, mobile RNAs with an established function or phenotype are still rare. Some of the best examples of these latter types include *StBEL5, -11, -29* [9,10] and *POTH1* [11] of potato, *CmGAI* of pumpkin [12], *PFP-LeT6* from tomato [13] and *AUX/IAA* [14], *FLOWERING LOCUS T* and *CENTRORADIALIS* [15,16,17] from *Arabidopsis*.

In addition to full-length mRNAs, small RNAs/miRNAs are also mobile through the plant’s phloem system and play important roles in both defense and development [18]. Numerous miRNAs have been identified in potato [19,20], and two of these, miR172 and miR156, have been implicated in the regulation of tuber formation [21,22]. It is conceivable that miRNAs of potato play an important role in regulating the activity of full-length, mobile mRNA signals [20].

### 1.2. Signals for Potato Tuberization 

Tuberization in potato is controlled by phloem-mobile signals that arise from the leaf. Based on previous work, the three most important signals that regulate the onset of tuber formation in potato are StCDF1, StSP6A and StBEL5 [9,27,28]. StCDF1 functions in the leaf with clock genes and the GI/FKF1 complex to mediate earliness [28]. StSP6A, an FT-like protein, and *StBEL5* mRNA are both mobile signals that originate in the leaf and under favorable conditions move down underground to the stolon tip to initiate tuber formation. We now understand that StBEL5 functions upstream to induce *StSP6A* activity, auto-regulates its own gene in stolons and induces numerous genes involved in the formation of the tuber [29]. POTH1 is a KNOTTED1-type TF that serves as a partner to StBEL5 and can also function as a mobile RNA [11]. The focus of this review, however, will be the novel group of mobile RNAs from the StBEL family that contribute to controlling tuber formation. BEL1- and KNOTTED1-type proteins are transcription factors from the three-amino-loop-extension (TALE) superfamily [30] that interact to regulate the expression of target genes. In potato, StBEL5 and its KNOX protein partner, POTH1, regulate tuberization by targeting genes that control the growth processes of the tuber pathway [29]. RNA movement assays demonstrated that *StBEL5* transcripts move through the phloem to stolon tips, the site of tuber induction. *StBEL5* mRNA originates in the leaf, and its movement to stolons is induced by a short-day (SD) photoperiod. This remarkable whole-plant communication system involves light induction of transcription in the leaf, photoperiod-activated mobilization of RNA and protein signals through the phloem and SD regulation of the promoters for both *StBEL5* and *StSP6A* in target organs growing underground in the dark [29,31].

### 1.3. The Tuberization Clade of the StBEL Family

**Figure 1:**
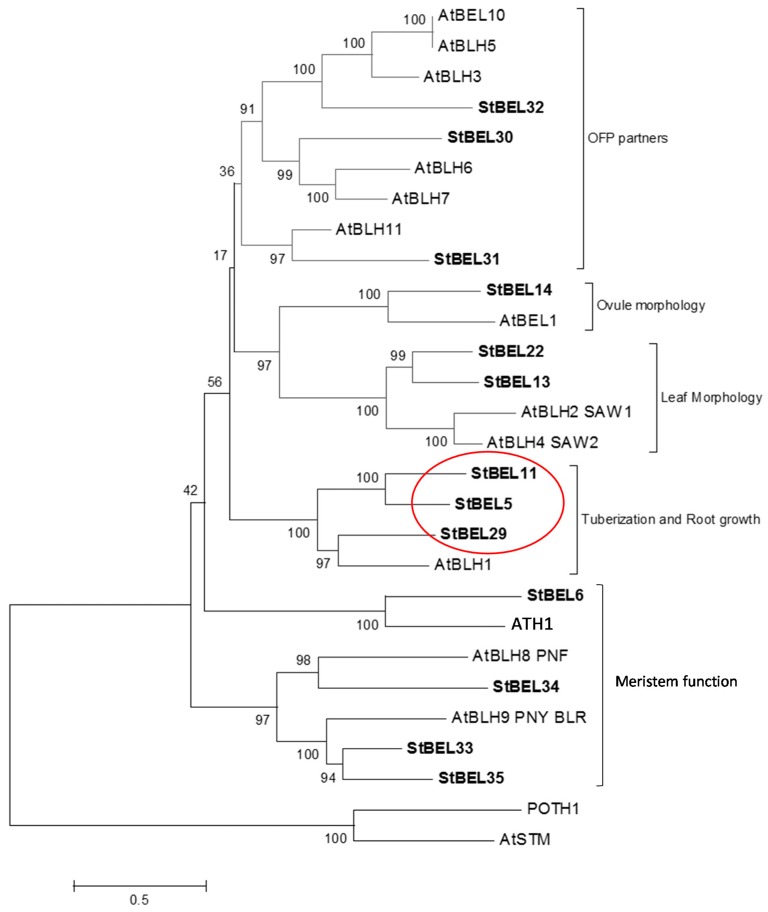
Phylogenetic relationship of the BEL1-like proteins of *Arabidopsis* and potato. The amino acid sequences of the thirteen known potato BEL1-like proteins were compared with BEL1 proteins of *Arabidopsis*. These data were organized into a phylogenetic tree with the MEGA4.0.2 package and the neighbor-joining program. The numbers listed at the branching points are boot-strapping values that indicate the level of significance (percentage) for the separation of two branches. The length of the branch line indicates the extent of difference according to the scale at the lower left-hand side. StBEL’s are represented in bold letters. Putative functions are listed for each group. The red oval designates the StBEL5 clade. OFP, OVATE FAMILY PROTEIN. With permission of the authors [39].

BEL1-like proteins are ubiquitous plant TFs that form heterodimers with KNOTTED1-type TFs and function in both floral and vegetative development [32,33,34,35]. Using the potato genome database, thirteen active BEL1-like genes have been identified each containing the conserved homeobox domain and the BELL domain, both of which are essential for the function of BEL1-type proteins [36,37]. Phylogenetic analysis of the StBEL family demonstrated a degree of orthology with the thirteen BEL1-like genes of *Arabidopsis*. Based on this analysis, StBEL proteins may be grouped into five main clades (Figure 1). One of these clades is designated the tuberization clade made up of *StBEL5*, *StBEL11* and *StBEL29.* These three StBEL types cluster with *AtBLH1* of *Arabidopsis* that functions with KNAT3 to affect the establishment of cell fates in the mature embryo sac [38]. Analyses of RNA abundance patterns using RNA-Seq data showed that these three *StBEL* genes make up approximately two-thirds of the total transcript values for the entire family [39]. Their RNA accumulation levels are very abundant in petioles and stems. Together, these observations suggest that *StBEL5*, *-11* and *-29* are relatively stable RNAs that play important roles in regulating development in actively-growing organs. All three exhibit enhanced levels of RNA accumulation in both leaves and stolons from short-day plants [39,40]. Within this group, *StBEL5* functions as a mobile RNA that impacts growth in both tubers and roots [9,31]. Because of their phylogenetic similarity and their overlapping RNA accumulation patterns, our initial assumption of this group was that its members were functionally redundant and potentially shared a similar long-distance, non-cell-autonomous delivery system. This review will update the functional roles of StBEL11 and StBEL29 and compare them to StBEL5’s regulatory role in controlling tuber formation.

## 2. Mobile RNAs of Potato

### 2.1. StBEL5 Functions as a Mobile RNA Signal

**Figure 2:**
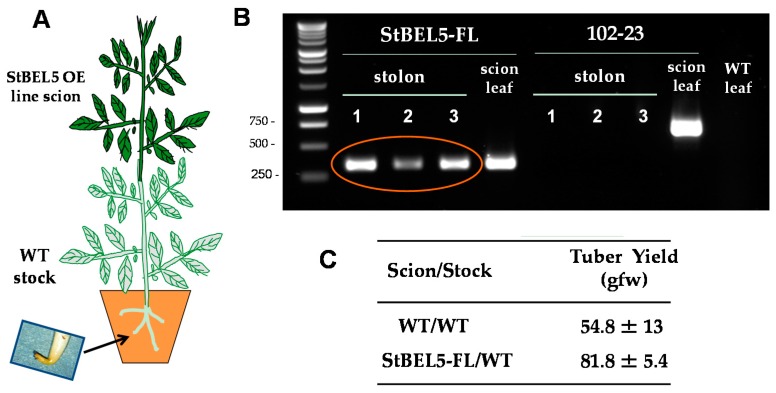
StBEL5 RNA moves through a heterograft graft, and this movement is associated with enhanced tuber yield. Soil-grown wild-type stock plants (*S. tuberosum* ssp. *andigena*) were grafted with scions from a StBEL5 overexpression line (**A**). After four weeks of long days (LDs) in the greenhouse, grafted plants were transferred to a growth chamber and acclimated under LD conditions for one week before transfer to short-day (SD) conditions. Leaf and stolon tip samples were harvested after 12 days of SD conditions and the RNA extracted. RT-PCR with gene-specific primers was performed (**B**) for both a negative control (a potato MADS box gene, 102-23) and test samples (StBEL5-FL, [full length]). Grafts made from an over-expression line for an antisense sequence of a potato MADS box gene (line 102-23) were used as a non-mobile control. RNA from scion leaf samples was used as a positive control (scion leaf). WT RNA from stolon tips, 0.5 cm in length, was sampled for both heterografts and used in the RT-PCR reactions. PCR was performed twice off a template made from RNA and reverse transcriptase. Two different gene-specific primers were used with a non-plant DNA tag specific for the transgenic RNA to discriminate from the native RNA. Three plants were assayed for both heterografts and are designated 1, 2 and 3. RNA from leaves of a WT/WT autograft was used as a negative PCR control (WT leaf lane). WT is non-transformed *andigena* line 7540. Similar negative results were obtained with RNA from autograft stolons. For tuber yields (**C**), plants were harvested after 28 days, and the mean of three plants was calculated for WT and StBEL5 grafted plants. WT scions grafted onto WT stocks were used as the yield controls. gfw, grams fresh weight. With permission of the authors [9].

**Figure 3:**
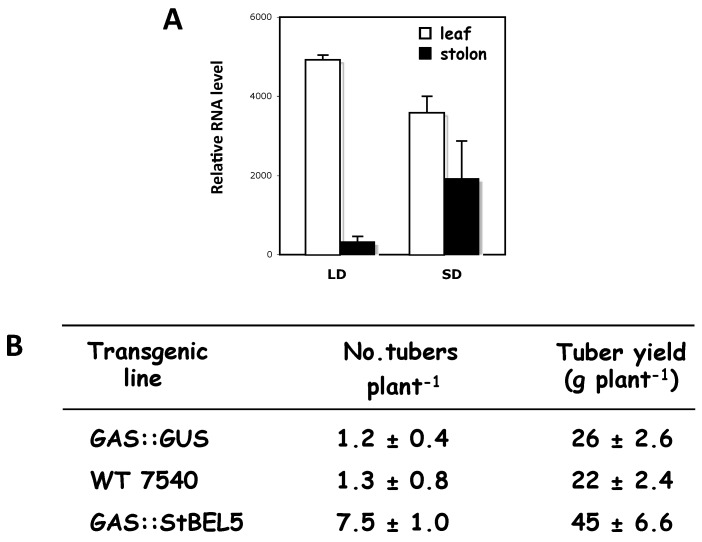
The correlation of *StBEL5* RNA movement into stolon tips (**A**) and tuber yields (**B**) in a transgenic line expressing full-length *StBEL5* transcripts driven by the leaf-specific *CmGAS* promoter [46,49]. A transgenic line with GUS transcripts driven by the same leaf-specific promoter was included as a control (**B**). Transgenic plants (*S. tuberosum* ssp. *andigena*) were grown under greenhouse conditions until the 12–14 leaf stage and then grown under short or long days for 10 days before harvest. Harvested plants were scored for tuber numbers after 10 days and tuber yields after 28 days under SD conditions (**B**). Total RNA was extracted from 0.5-cm stolon tips and new leaves from three separate plants. RT-PCR was performed using a non-plant sequence tag fused to all transgenic RNAs, and a gene-specific primer from the 3′ UTR of St-*BEL5* (**A**). The full-length *BEL5* and GUS constructs were cloned downstream from the *CmGAS* promoter in the binary vector, pBI101.2. Non-transformed control plants are designated “WT 7540”. Homogenous PCR products were quantified (**A**) by using ImageJ software and normalized by using rRNA values [9]. Standard errors of the means of the three biological replicates are shown (**A**,**B**). □, leaf; ■, stolon (**A**). GUS expression was detected in leaves, but not stolons of GAS::GUS transgenic lines of *S. tuberosum* ssp. *andigena* [46]. With permission of the authors [9].

The two principal factors that function as long-distance mobile signals for controlling the onset of tuber formation are the FT ortholog, StSP6A [27,41], and the full-length mRNA of *StBEL5*. Both arise from the leaf and under inductive conditions are transported via the phloem underground to the stolon tip to initiate tuber formation [42,43]. StBEL5 of potato functions with a KNOX partner to regulate tuber growth [9,37,40]. In plants, BEL1-like proteins form a complex with KNOX-type transcription factors [36,39,44] to regulate numerous aspects of growth and development [32,33,34,40,45]. This BEL/KNOX complex recognizes a specific *cis*-element, a double TTGAC motif present in target genes of BEL1-like TFs. StBEL5 is unique in that its full-length transcript has the capacity to move long distances through the sieve element system [9,31,46]. Long-distance transport of *StBEL5* mRNA through the sieve element system has been verified using RNA movement assays in both heterografts (Figure 2) and whole plants (Figure 3). Heterografts have featured a transgenic scion grafted onto a non-transformed WT *S. tuberosum* ssp. *andigena* line 7540 stock. With such a graft, movement of transgenic *StBEL5* RNA, or any potential mobile RNA, may be tracked downward across the graft union (Figure 2A,B). To map *StBEL5* RNA movement using another model system, the leaf-specific galactinol synthase (GAS) promoter was employed (Figure 3). Utilizing this system coupled with RT-qPCR, RNA mobility to other parts of the plant may readily be monitored. In both cases, enhanced mobility of *StBEL5* is strongly correlated to increased tuber yields (Figure 2C and Figure 3B). Movement of StBEL5 is enhanced by SDs (Figure 3A) and is mediated by select RNA-binding proteins [47]. Expression of *StBEL5* is activated in leaf veins and petioles by low levels of blue and red light, but not by day length [48]. 

In a process that leads to tuber induction, StBEL5 enhances its own activity in stolons and augments StSP6A activity in both leaves and in newly tuberizing stolon tips [29,31]. Expression of both *StBEL5* and *StSP6A* is strongly induced in stolons under short-day conditions by the long-distance transport of *StBEL5* signal RNA [29]. In both overexpression and suppression lines of StBEL5, accumulation of *StSP6A* was tightly correlated with the activity of StBEL5. By mutating five *cis*-elements specific for StBEL5 binding in the upstream genomic sequence of *StSP6A*, fusing these to a GUS marker and analyzing the construct in transgenic lines, SD-induced transcriptional activity of StSP6A was eliminated [29]. Overall, these results strongly suggest that *StSP6A* is a transcriptional target of StBEL5 in both leaves and stolons and help to elucidate the mechanism for the “autoregulatory loop” formulated to explain SD-induced accumulation of transcripts of *StSP6A* in stolons [27]. Through this double induction of StSP6A in leaves and stolons and the auto-regulation of its own gene in stolons, StBEL5 functions to directly activate the tuberization program and to amplify the StSP6A signal. 

There are numerous reports of StBEL5’s positive effect on tuberization [9,40,46,47]. Overexpression (OE), enhanced accumulation and regulated movement of its mRNA led to early, increased tuber yields by as much as five-fold in both the photoperiod-responsive subspecies *S. tuberosum andigena* and the commercial cultivar, Désirée. Despite these yield increases, shoot growth and morphology were normal, suggesting that the effect of StBEL5 OE on overall plant growth is specific. Tuber morphology was also similar to WT. Consistent with these results, RNA suppression of StBEL5 resulted in drastic reductions in tuber yields [29,47]. In summary, careful analysis of StBEL5 activity using both OE and RNA suppression lines has established a very robust correlation between StBEL5 accumulation and potato tuber yields. Supporting this role in tuberization, recent analysis of the StBEL5-induced transcriptome identified thousands of targets genes, many of them involved in growth processes occurring in the newly-forming tuber [29]. Examples of StBEL5-target genes that are involved in tuberization include *GA2 OXIDASE1* [50], *GA20 OXIDASE1* [51], *LONELYGUY* [52], *AGL8* [53], the *PINs* [54], *AUX/IAA* [55] and *POTLX1* [56]. In addition, expression and promoter analyses have shown clearly that StBEL5 activates three critical tuber signals, its own gene through auto-regulation [31], the aforementioned StSP6A and the leaf-specific signal, StCDF1 [29]. Developmental studies with StBEL5 overexpression and suppression lines demonstrated a very strong positive correlation between StBEL5 and *StSP6A* gene activity [29]. It is through this overall pattern of transcriptional control and its targeted RNA mobility that StBEL5 affects its impact on tuber formation [9,29].

### 2.2. Mobility and Function of StBEL11 and StBEL29

**Figure 4:**
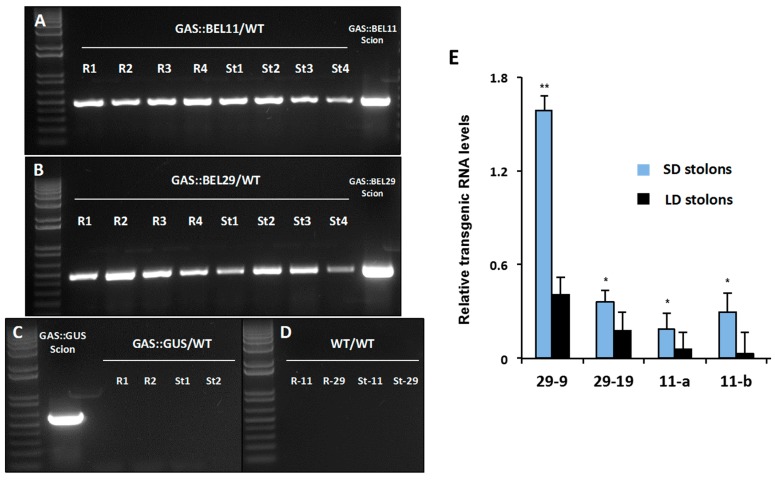
Movement of transgenic *StBEL11* (**A**) and *-29* (**B**) mRNA across heterografts of soil-grown plants. Heterografts were performed with four replicates of *GAS::BEL11* (line 11b), *GAS::BEL29* (line 29-9) and *GAS::GUS* scions on non-transformed wild-type *S. tuberosum* ssp. *andigena* line 7540 (WT) stocks. Culture conditions and one-step RT-PCR using transgenic-specific primers were previously described [10]. All PCR products detected in scion (positive control) and stock (test for movement) RNA samples represent transgenic RNA. RNA from scion leaf samples was used as a positive control (scion samples). Heterografts are designated R1-4 (root stock RNA) and St1-4 (stolon stock RNA). Negative controls for movement of GUS (**C**) and PCR of WT (**D**) RNA are included. Photoperiod effect on the movement of transgenic *StBEL29* and *StBEL11* mRNA from leaves into stolons was assessed (**E**). Full-length transgenic RNA was driven by the leaf-specific GAS promoter of melon [46,49]. GAS::BEL11 and -29 plants were grown under either long-day (LD) or short-day (SD) conditions [10]. RT-qPCR with gene-specific primers was used to calculate the amount of transgenic RNA in stolons. Samples were measured in duplicate and normalized against *StActin8* mRNA. The fold change in RNA levels was calculated as the 2^−ΔΔCt^ value relative to transgenic RNA (set at a value of 1.0) detected in the source leaf. Standard errors of the means of two biological replicates are shown with one or two asterisks indicating a significant difference (*p* < 0.05 or *p* < 0.01, respectively). With permission of the publisher [10].

Because of their close sequence matches, including highly conserved functional domains and accumulation in stolons during the onset of tuber formation, we considered the possibility that members of the StBEL5/StBEL11/StBEL29 clade of StBEL-like TFs share both the mode of action and function in relation to tuber development. For example, the promoters of all three are active in leaf vasculature [9,10]. More specifically, do StBEL11 and -29 exhibit mobility of their RNAs through the phloem? Are they redundant for a tuberization function? Making use of heterografts and transgenic lines that tag the target RNAs with non-plant sequence and that limit their source expression to leaf veins, experiments were designed to test the long-distance transport capacity of *StBEL11* and *StBEL29*. Several replicate heterografts of transgenic scions grafted onto WT stocks clearly demonstrated that both RNAs moved from leaves across the graft union into roots and stolon tips (Figure 4A,B), whereas control RNAs did not (Figure 4C,D). Using transgenic plants that drive *StBEL11* and *-29* expression by the leaf-specific GAS promoter [49], movement of the transgenic RNAs into stolon tips was enhanced by SD conditions (Figure 4E). The GAS promoter is specific to minor veins of the leaf mesophyll and so is an excellent promoter to use to assess movement through the leaf vasculature. In potato, the GAS promoter is active in the minor veins of leaves, and its activity was not detected in roots, stolons or tubers [46]. Because of the organ-specific activity of the GAS promoter, only transgenic RNA that was transported from the leaf was quantified in stolons (Figure 4E). These movement dynamics are similar to StBEL5 [9,46]. In summary, the three sequence-related StBEL’s, StBEL5, -11 and -29, are the only BELs of potato with RNA levels that increase in response to short-day conditions and that have been confirmed to be phloem-mobile [9,10,40].

**Figure 5:**
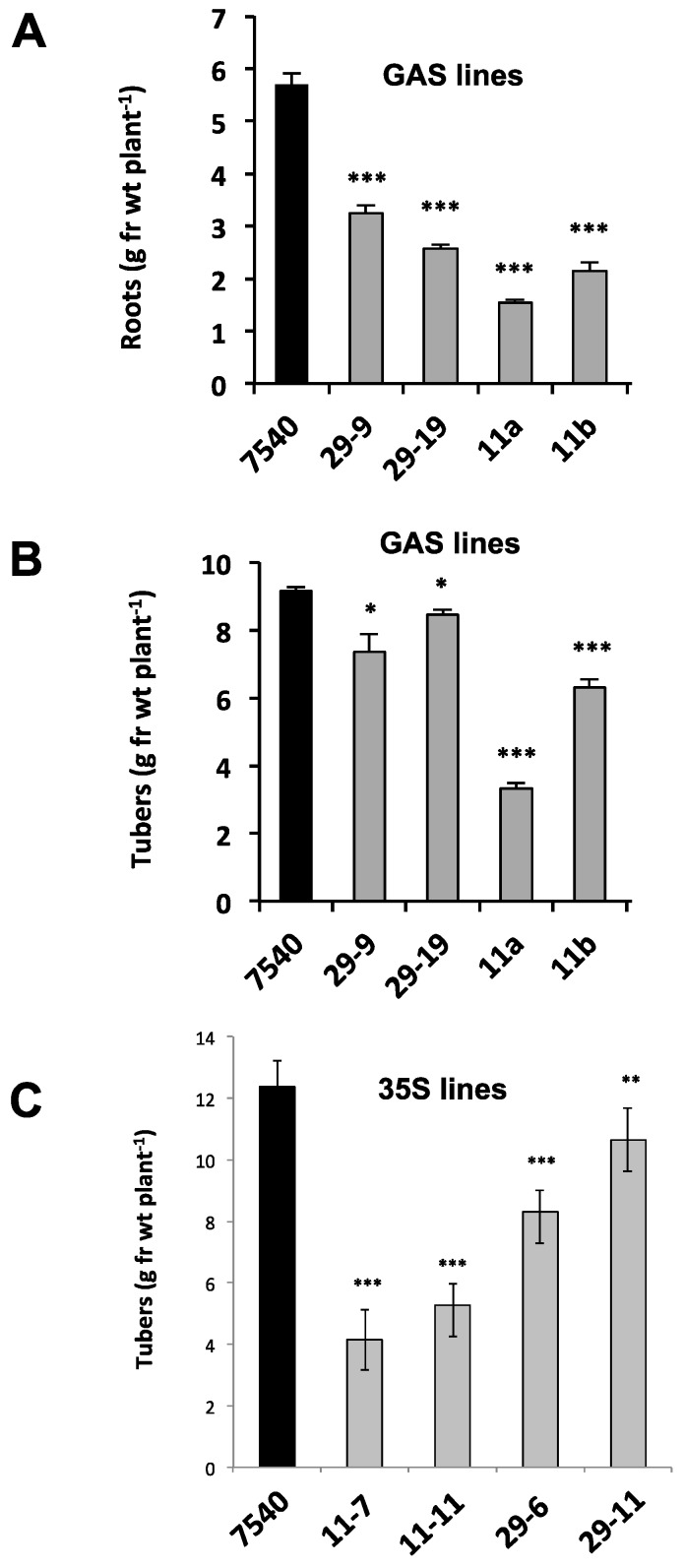
Root (**A**) and tuber (**B**) yields from non-transformed *S. tuberosum* ssp. *andigena* (WT line 7540) and GAS::StBEL11 and -29 transgenic lines grown under short-day conditions in growth chambers. (**C**) Tuber yields in 7540 (WT), 35S::StBEL11 (lines 7 and 11) and 35S::StBEL29 (lines 6 and 11) transgenic lines. Plants were grown under long days for four weeks followed by three (GAS lines) or four (35S lines) weeks under short-day conditions. At harvest, shoots were weighed, and tubers and roots were washed in water, blotted dry and weighed. Data (g·fr·wt plant^−1^ = grams fresh weight per plant) represent the mean of four (GAS lines) or seven (35S lines) biological replicates. Error bars represent ±SD. One, two or three asterisks indicate significance (*p* < 0.05, *p* < 0.01 and *p* < 0.001, respectively) using a Student’s *t-*test. By permission of the publisher [10].

**Figure 6:**
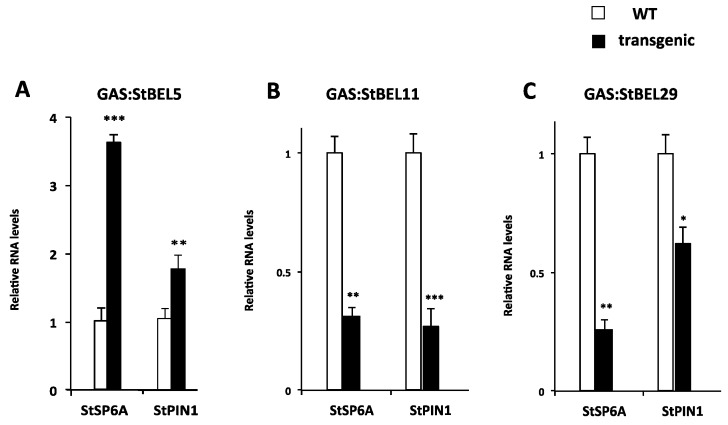
Relative levels of *StSP6A*, and *StPIN1* RNA in tuberizing stolons of WT 7540 *andigena* (open bars), GAS:StBEL5 (**A**), GAS:StBEL11 (**B**) and -29 (**C**) transgenic lines (black bars) grown under short-day conditions. Data represent the mean of two biological replicates and two technical reps. Plants were grown under long days for four weeks followed by two weeks under short-day conditions. StBEL5 enhances the levels of RNA for these target genes whereas, StBEL11 and -29 suppress the activity of these target genes. With permission of the publisher and authors [10,29].

**Figure 7:**
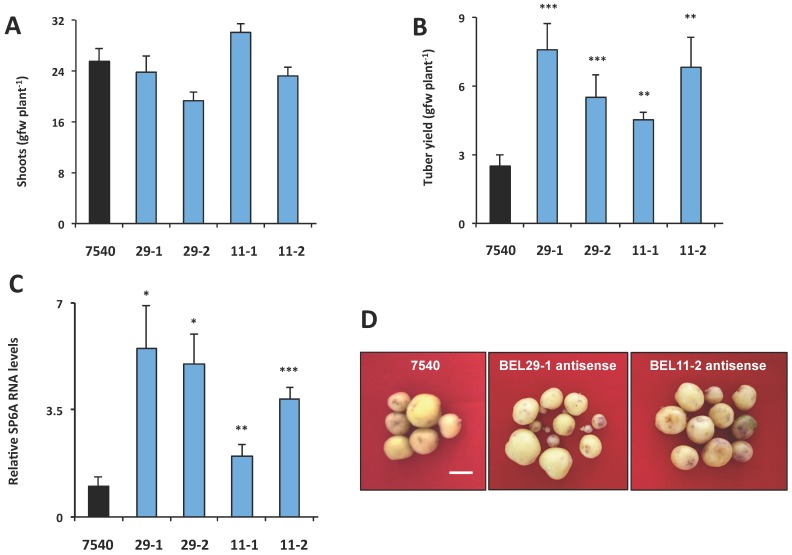
Phenotype of RNA antisense lines for StBEL11 (11-1, 11-2) and StBEL29 (29-1, 29-2). After screening, select independent lines were grown for eight weeks under long days and then 21 d under SD conditions, harvested and scored for shoot growth (**A**) and tuber yields (**B**). *Andigena* line 7540 is a non-transformed WT line. For analysis of the tuber target gene, *StSP6A* (**C**), RNA was extracted from 21-day SD stolons and quantified using RT-qPCR with gene-specific primers. Error bars represent ±SD of five plants for the whole plant analysis and two biological replicates for the RT-qPCR. A Student’s *t*-test was performed to check significance with one, two and three asterisks indicating *p*-values of <0.05, <0.01 and <0.001, respectively. Tuber samples from RNA antisense lines for StBEL11 and StBEL29 (**D**). Plants were grown in a growth chamber as described above prior to harvest. For each line, tubers were pooled from three plants. White bar = 1.0 cm. With permission of the publisher [10].

To determine if this phloem mobility was related to tuber development, transgenic lines were again utilized to assess the function of StBEL11 and StBEL29. Several transgenic lines that overexpressed *StBEL11* or *StBEL29* were examined utilizing both the leaf-specific GAS promoter (Figure 5A,B) and a CaMV 35S promoter (Figure 5C). Despite very little change in shoot growth [10], OE in these GAS:BEL lines led to a reduction in both root and tuber growth (Figure 5A,B), whereas OE using the CaMV 35S promoter resulted in decreased tuber yields (Figure 5C). In direct opposition to the enhanced effect mediated by OE of StBEL5 (Figure 6A), GAS:BEL lines of both *StBEL11* and *StBEL29* suppressed RNA levels of the tuber marker genes, *StSP6A* and *StPIN1* (Figure 6B,C). To round out this analysis, transgenic lines that suppressed *StBEL11* and *StBEL29* through an antisense strategy were employed [10]. In this case, despite negligible changes in shoot growth, suppression of both of these types led to enhanced tuber yields (Figure 7A,B). Concomitant with this yield increase, RNA levels of the tuber signal gene, *StSP6A*, increased by as much as five-fold in these transgenic lines (Figure 7C), whereas in StBEL5 suppression lines, *StSP6A* RNA levels were reduced [29].

Tubers from the StBEL11 and -29 antisense lines appeared to exhibit a normal morphology (Figure 7D). Based on the results from both OE and suppression lines, it is likely that these tuber phenotypes are caused by a change in the tuber genetic program. In the case of StBEL5, OE leads to an enhancement of yield, whereas OE of StBEL11 or -29 suppresses tuber yields [10,40,46]. Considering the high degree of conservation in the amino-acid sequences of the functional domains of these three StBEL types [39], it is not surprising that they mediate the activity of some of the same target genes. 

## 3. Mechanism for *StBEL* RNA Movement: The Role of the PTB Proteins 

Although there are numerous reports of full-length mobile RNAs in plants, there is very little information on the RNA sequence that mediates this process [12,57]. Such sequences, designated zip code elements, have been identified in the RNAs of animals and function in an interaction with RNA-binding proteins to control location and stability [58,59,60]. Most often, these zip code sequences are located in the 3′ untranslated regions (UTR) of the transcript. Recently, several papers have reported on the role of important RNA-binding proteins that facilitate the movement and stability of phloem-mobile RNAs in both pumpkin and potato [24,47].

**Figure 8:**
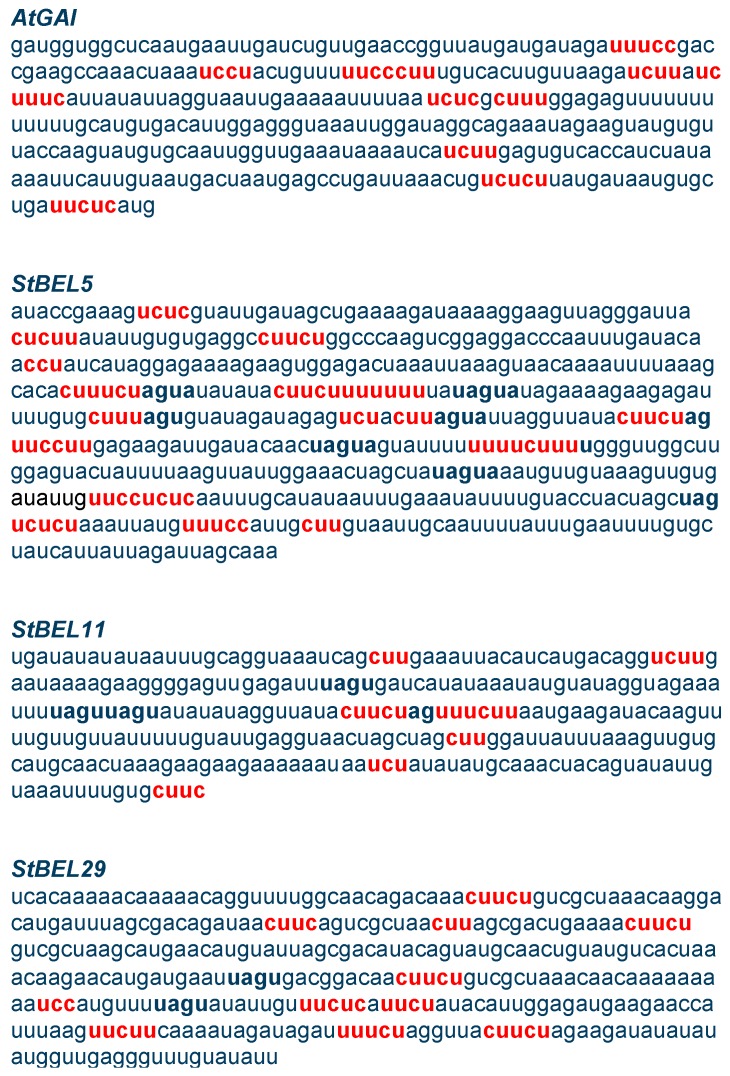
Cytosine/uracil (CU) motifs present in the 3′ UTRs of four mobile RNAs, *AtGAI* [57], *StBEL5* [9] and *StBEL11* and -*29* [10]. CU motifs of three nt or more are designated in a bold red font. GenBank accession numbers are Y15193 for *AtGAI*, AF406697 for *StBEL5*, AF406698 for *StBEL11* and AF406702 for *StBEL29*. These CU motifs are putative targets for the four RNA-recognition motifs present in polypyrimidine tract-binding proteins that bind numerous RNAs to regulate their metabolism [47,65].

In both cases, the key RNA-binding proteins involved were polypyrimidine tract-binding (PTB) proteins. These proteins contain four RNA recognition motifs that bind at four cytosine/uracil (CU) motifs located within 100–200-nt regions inside the UTRs of the target RNAs [61]. In the case of *StBEL5*, the CU-rich sequence present in its 3′ UTR (Figure 8) facilitates binding to two RNA-binding proteins, designated StPTB1 and StPTB6 (*Solanum tuberosum* polypyrimidine tract-binding protein) [47]. Previous work has confirmed that these PTB proteins fine-tune and optimize StBEL5 activity during tuber formation by enhancing the movement, stability and activity of the *StBEL5* RNA [47]. Movement assays using a PVX vector system suggest that, similar to StBEL5, the StPTB proteins are mediating movement of *StBEL11* and *StBEL29* [10]. Because of StBEL5’s importance as a TF and its significant effect on development [29], and similar to other biological systems [62,63,64], the location and timing of StBEL5 activity is extremely critical for coordinating cell growth. Promoter analysis of the two StPTB’s and StBEL5 demonstrates their strong concordant spatial and temporal patterns of expression, particularly in vascular tissue [9,47]. All three have expression associated with phloem cells and newly-formed tubers. In independent transgenic lines, overexpression and suppression of StPTB1 and StPTB6 are directly correlated to the movement and stability of *StBEL5* RNA [47]. 

The thirteen BEL RNAs of potato exhibit a wide range of RNA accumulation patterns [39]. *StBEL5*, *-11* and *-29* are by far the most abundant RNAs in the StBEL family and the only ones confirmed to be phloem-mobile [9,10,31]. The length of the 3′ UTRs of these three mobile RNAs are the longest of the group at 505, 317 and 491 nt, respectively. Within the transcript sequence of all three, CU motifs are repeated several times in the UTRs (Figure 8). The 3′ UTRs of *StBEL5*, *StBEL11* and *StBEL29* contain 16, 7 and 11 CU motifs, respectively (Figure 8, red highlighted nucleotides). *AtGAI*, a confirmed mobile RNA of *Arabidopsis*, contains 10 CU motifs in its 3′ UTR (Figure 8). These uracil/cytosine-rich motifs very likely mediate the binding of the RNA to the StPTB proteins [24,47]. By way of comparison, the non-mobile *StBEL* mRNAs, *StBEL14* and *-22*, have three and two CU motifs, respectively, in their 3′ UTRs. Considering that the mobility of all three of these RNAs was enhanced under an SD photoperiod (Figure 2, Figure 3 and Figure 4), it is feasible that all three mobile RNAs are transported by the same RNA/protein complex.

## 4. Final Perspectives

How does the repressive activity of StBEL11 and -29 work within the framework of tuber development? Could these mobile tuber signals mediate cell-specific control of growth in coordination with StBEL5 activity? The nascent tuber evolves from a specific cell layer within the apical meristem of the stolon and is characterized by a change in the direction of cell growth from transverse divisions and cell elongation to longitudinal cell division [66]. The apical portion of the stolon meristem becomes dormant soon after tuber initiation. At the onset of tuber formation, after stolon elongation has ceased, a band of cells within the pith and cortex enlarges and divides longitudinally. This results in swelling in the stolon tip that spreads throughout the subapical portion of the meristem [66]. Further size increases are mediated by the development of cells within the perimedullary region located between the pith and the cortex. These bands of progenitor cells are connected to the vascular tissue and so would be readily accessible to any mobile signals traveling along the sieve element system and into the stolon tip [66]. Locally, these processes are regulated by changes in hormone levels [67]. For example, the radial swelling that occurs in the subapical meristem of the stolon is caused by a reduction in gibberellic acid (GA) levels that leads to a reorientation of cortical microtubules to a longitudinal direction [68,69]. Under high levels of GA, the stolon tip elongates, and as GA levels drop dramatically in the developing tuber, cell division is more randomly aligned [67]. This results in a globular-shaped organ, the tuber, that will eventually become more ovate in shape. Other hormones, such as auxin and cytokinins, also play important roles in controlling tuber formation [52,70,71]. Therefore, during the onset of tuberization, some cells in the stolon meristem become very active, whereas others in close proximity remain dormant. Analyses of the transcriptional targets of StBEL5 (and other TALE TFs) indicate that a large proportion of these are involved in hormone metabolism [29,72]. For example, StBEL5 suppresses GA levels in stolons by regulating the transcription of important genes controlling GA metabolism [29,31,37]. Through similar transcriptional controls, cytokinin levels are enhanced [29,40]. StBEL11/29 and StBEL5 working in tandem with their KNOX partners may readily function as stop-and-go switches that tightly maintain a balance in cell growth as the incipient tuber takes form. This process is comparable to the classic florigen/anti-florigen model [73]. In this system, flowering locus T (FT) protein acts as the floral signal that moves into the apex and binds to the basic leucine zipper TF, FD, to induce flowering. TERMINAL FLOWER 1-like proteins function as floral inhibitors and are antagonistic to FT function. As little as a single amino-acid change in the FT protein sequence was sufficient to transform it from a floral activator to a floral repressor [74].

In summary, previous work indicates that the full-length mRNAs of *StBEL5*, *StBEL11* and *StBEL29* are phloem-mobile and that this movement is enhanced by SD and may be regulated by a common mechanism. Enhanced movement and accumulation of *StBEL5* RNA results in a phenotype characterized by increased earliness and tuber yields. The movement and stability of its transcript is mediated by an interaction with the RNA-binding proteins, StPTB1 and StPTB6. *StBEL11* and *StBEL29* regulate tuber formation, but they function in opposition to the growth-promoting features of StBEL5. They both appear to inhibit growth by targeting select genes that are involved in tuber development. Consistent with this premise, suppression of both *StBEL11* and *-29* specifically increases tuber yields. In creating a complex signaling pathway, potato has evolved a long-distance transport system that regulates underground organ development through full-length mRNAs that function as both activators and repressors. The three StBEL’s of the tuberization clade appear to balance tuber growth by mobilizing their mRNAs through an interaction with RNA-binding proteins. StBEL5 functions to directly activate the tuberization program and to amplify other important signals in the pathway [29]. In this model, StBEL11 and -29 function antagonistically in this process to repress tuber growth. 

## Figures and Tables

**Table 1:** Phloem-mobile mRNAs of transcription factors that move across heterografts.

RNA	Annotation	Putative Function	Reference
*MpSLR/IAA14*	Auxin response factor	Transcriptional repressor	[23]
*CmSCL14P*	Scarecrow-like	Transcription factor	[24]
*CmSTM*	Shoot meristemless	Meristem regulator	[24]
*CmERF*	Ethylene response factor	Ethylene signaling	[24]
*CmNAC*	NAM, ATAF1/2 and CUC2	Meristem development	[25]
*CmMyb*	Myb-like transcription factor	Transcriptional activator	[24]
*BoFVE*	Mammalian retinoblastoma-associated protein	Floral regulator	[26]
*BoAGL24*	Agamous-like	Floral regulator	[26]
AtAux/IAA18 and -28 *	Auxin response factor	Auxin signaling ⇩	[14]
CmGAI *	GA Insensitive	Leaf morphology ⇧	[12]
StBEL5 *	Potato BEL1-like family	Tuber growth ⇩	[9]
StBEL11/29 *	Potato BEL1-like family	Tuber growth ⇩	[10]
POTH1 *	Potato KNOTTED1-type	Vegetative growth ⇩	[11]
PFP-LeT6 *	Tomato Knotted1-type fusion	Leaf morphology ⇧	[13]
FT *	Arabidopsis Flowering locus T	Activates flowering ⇧	[15]
ATC *	Arabidopsis CENTRORADIALIS	Represses flowering ⇧	[16]

* Indicates that movement of the RNA is associated with a phenotype. *At*, *Arabidopsis thaliana*; *Cm*, *Cucurbita maxima*; *Le*, *Lycopersicon esculentum*; *Mp*, *Malus prunifolia*; *Bo*, *Brassica oleracea*; *St*, *Solanum tuberosum*; PFP, pyrophosphate-dependent fructose 6-phosphate phosphotransferase. Arrows in the function column of the last seven RNAs indicate the prominent direction of the mobile transcript through a graft union.
